# Chromosome-level assembly and annotation of the *Xyrichtys novacula* (Linnaeus, 1758) genome

**DOI:** 10.1093/dnares/dsad021

**Published:** 2023-10-05

**Authors:** Fernando Cruz, Jèssica Gómez-Garrido, Marta Gut, Tyler S Alioto, Joan Pons, Josep Alós, Margarida Barcelo-Serra

**Affiliations:** Centro Nacional de Análisis Genómico (CNAG), C/Baldiri Reixac 4, 08028 Barcelona, Spain; Centro Nacional de Análisis Genómico (CNAG), C/Baldiri Reixac 4, 08028 Barcelona, Spain; Centro Nacional de Análisis Genómico (CNAG), C/Baldiri Reixac 4, 08028 Barcelona, Spain; Centro Nacional de Análisis Genómico (CNAG), C/Baldiri Reixac 4, 08028 Barcelona, Spain; Institut Mediterrani d’Estudis Avançats, IMEDEA (UIB-CSIC), C/Miquel Marquès 21, 07190 Esporles, Illes Balears, Spain; Institut Mediterrani d’Estudis Avançats, IMEDEA (UIB-CSIC), C/Miquel Marquès 21, 07190 Esporles, Illes Balears, Spain; Institut Mediterrani d’Estudis Avançats, IMEDEA (UIB-CSIC), C/Miquel Marquès 21, 07190 Esporles, Illes Balears, Spain

**Keywords:** genome annotation, chromosome-level assembly, Hi-C scaffolding, Labridae, Xyrichtys novacula

## Abstract

The pearly razorfish (*Xyrichtys novacula*), commonly known as *raor* in the Balearic Islands, is a wrasse within the family Labridae. This fish species has particular biological and socio-cultural characteristics making it an ideal model organism in the fields of behavioural ecology, molecular ecology and conservation biology. In this study, we present the first annotated chromosome-level assembly for this species. Sequencing involved a combination of long reads with Oxford Nanopore Technologies, Illumina paired-end short reads (2 × 151 bp), Hi-C and RNA-seq from different tissues. The nuclear genome assembly has a scaffold N50 of 34.33 Mb, a total assembly span of 775.53 Mb and 99.63% of the sequence assembled into 24 superscaffolds, consistent with its known karyotype. Quality metrics revealed a consensus accuracy (QV) of 42.92 and gene completeness > 98%. The genome annotation resulted in 26,690 protein-coding genes and 12,737 non-coding transcripts. The coding regions encoded 39,613 unique protein products, 93% of them with assigned function. Overall, the publication of the *X. novacula*’s reference genome will broaden the scope and impact of genomic research conducted on this iconic and colourful species.

## 1. Introduction

The pearly razorfish (*Xyrichtys novacula*), commonly known as *raor* in the Balearic Islands is a marine teleost benthic fish species belonging to the family Labridae (wrasses). The species is distributed in the Mediterranean and along the temperate coasts of the Atlantic Ocean, including the Caribbean Sea.^1,2^ Its diet primarily consists of small crustaceans and echinoderms,^3,4^ which are commonly found in its preferred habitat. These habitats typically comprise sandy and muddy bottoms surrounded by seagrass meadows allowing for the species’ particular burrowing behaviour in the sand which serves as a protection when threatened or at night for rest.^5^ Individuals of *X. novacula* are protogynous hermaphrodites, initially developing as females and later transitioning to males. This reproductive system commonly gives rise to a harem-like social structure,^6,7^ where a male defends its territory encompassing several smaller female territories, usually up to six in this species.^8–10^

In the Balearic Islands, this species has a long-standing history of being harvested in local fisheries and highly valued in markets, with records of this tradition dating back to the 1500s.^11^ Its capture is mainly limited to traditional methods, such as hook and line gear. Nevertheless, it remains a species heavily harvested by recreational fishers, with up to 60% of the population being captured annually in heavily harvested areas of the Balearic Islands.^12^ In this area, population management has primarily focussed on population density and closed seasons during reproduction, which seem to maintain population numbers over time.^12^ However, the genetic and genomic consequences of this potentially selecting pressure have not been studied.

Beyond its economic and cultural importance, the biological peculiarities of *X. novacula* makes it an ideal model species for studies in behavioural, molecular and conservation ecology. In particular, its reproductive biology is of special interest because of its apparent lack of sexual chromosomes and its sex transition likely regulated by environmental factors, a phenomenon also observed in other species with similar characteristics.^13^ Although research has been conducted on species with comparable sex determination processes, we still do not have a comprehensive understanding of the specific molecular mechanisms at the genomic, epigenetic and gene regulation levels.^14,15^ Regarding behavioural ecology, this species’ behaviour can be easily quantified in the laboratory and in the wild,^10,16^ enabling the exploration of genotype-phenotype associations that shed light on molecular mechanisms underlying ecologically relevant behaviours. For instance, research has shown that certain behavioural types, including chronotypes,^17^ can be more vulnerable to capture by fishers, providing an opportunity to study the evolutionary consequences of trait selective harvesting on exploited fish populations.^12,18^ Moreover, it has been suggested that marine protected areas can act as reservoirs for vulnerable genotypes,^19^ ensuring the maintenance of genetic variability in local resident populations of species like *X. novacula*.

In this study, we sequenced, assembled and annotated the complete genome of *X. novacula* using an individual collected from the Mediterranean Sea (Balearic Islands). The resulting chromosome-level genome assembly has 24 superscaffolds supporting the findings reported by Vitturi et al.^20^ and Almeida et al.,^21^ which also found a diploid chromosome number of 48 for this species (But see,^22,23^ reporting different chromosome numbers in Caribbean populations, suggesting the presence of potentially different cryptic species within the *X. novacula* range). The results from this work represent a significant advancement in the genomic research on this iconic species, positioning it as a potential model species to disentangle the ecological and evolutionary impact of exploitation on small coastal fish subject to localized but intense fishing pressures.^12^

## 2. Materials and methods

### 2.1 Sample collection and processing

**Figure 1.**
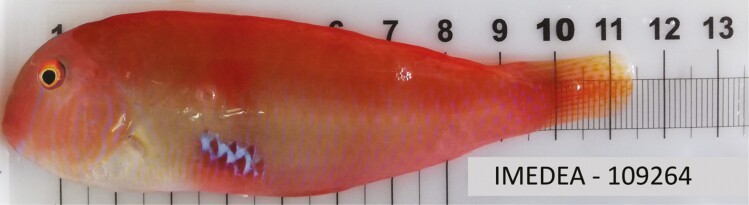
*Xyrichtys novacula.* Specimen of *X. novacula* used to generate the genome (ID: XN0914), deposited at the Mediterranean Institute for Advanced Studies Collection (Ref. IMEDEA 109264). Scale bar in cm.

An individual of *X. novacula* (ID: XN0914) was captured in the Marine Protected Area of the Palma Bay (39.47568°N, 2.72149°E) in September 2021 using the standard capture methods for this species, hook and line gear. This individual was kept in captivity for behavioural assays and euthanized using an overdose of 1 g^−1^ solution of tricaine methanesulfonate (MS-222). Sampling and experimental permits were granted by the Ethics Committee of Animal Experimentation of the University of the Balearic Islands and the Balearic Government (Ref. CEEA 107/01/19). Liver, brain, spleen and muscle tissue were immediately dissected and fresh-frozen in liquid nitrogen. Samples were stored at −80°C until DNA extraction. The individual (Fig. 1) and remaining frozen tissue at −80°C were deposited at the Mediterranean Institute for Advanced Studies Collection (Ref. IMEDEA 109264).

### 2.2. Genomic DNA extraction and sequencing

To extract high molecular weight (HMW) genomic DNA (gDNA), the Nanobind tissue kit (Circulomics) was used with a fresh frozen liver sample following the manufacturer’s protocol. The HMW gDNA eluate was quantified by Qubit DNA BR Assay kit (Thermo Fisher Scientific) and the DNA purity was evaluated using Nanodrop 2000 UV/Vis (Thermo Fisher Scientific) measurements. To determine the gDNA integrity Femto Pulse (Agilent) Genomic DNA 165 kb kit (Agilent) was used. The gDNA samples were stored at 4°C.

#### 2.2.1 Genomic long reads

HMW gDNA was subjected to quality control for purity, quantity and integrity in preparation for long-read sequencing. The sequencing libraries were prepared using the 1D Sequencing kit SQK-LSK110 from Oxford Nanopore Technologies (ONT). In brief, 3.0 μg of the gDNA underwent end-repair and adenylation using the NEBNext UltraII End Repair/dA-Tailing Module (NEB), followed by ligation of sequencing adaptors. The ligation product was purified using 0.4X AMPure XP Beads and eluted in Elution Buffer (ONT).

The sequencing run was performed on a PromethION 24 instrument (ONT) using a flow cell R9.4.1 FLO-PRO002 (ONT), and the sequencing data was collected for 110 h. The quality parameters of the sequencing runs were monitored in real time using the MinKNOW^TM^ platform v21.11.7, and the basecalling was performed using Guppy v5.1.13.

#### 2.2.2 Genomic short reads

The short-insert paired-end (PE) libraries for whole genome sequencing were prepared using the polymerase chain reaction (PCR)-free protocol and the KAPA HyperPrep kit (Roche). After end-repair and adenylation, Illumina platform-compatible adaptors with unique dual indexes and unique molecular identifiers (Integrated DNA Technologies) were ligated. The sequencing libraries were quality controlled on an Agilent 2100 Bioanalyzer using the DNA 7500 assay (Agilent) to assess size and quantified using the Kapa Library Quantification Kit for Illumina platforms (Roche). Libraries were sequenced on Illumina NovaSeq 6000 with a read length of 151 bp PE following the manufacturer’s protocol for dual indexing. Image analysis, base calling and quality scoring of the run were processed using the manufacturer’s software Real Time Analysis (RTA) v3.4.4.

### 2.3 Proximity ligation, library preparation and sequencing

For the proximity ligation library, the Omni-C kit (Dovetail) was used with fresh-frozen spleen as starting material following the manufacturer’s protocol. After reversal crosslinking, the DNA was purified and followed by the preparation of Illumina-compatible PE sequencing libraries. Biotinylated chimeric molecules were isolated using streptavidin beads before PCR enrichment of the library. The library was amplified with 12 PCR cycles using KAPA HiFi HotStart Ready Mix (Roche) and sequenced on Illumina NovaSeq 6000 (151 bp PE) following the manufacturer’s protocol for dual indexing. Image analysis, base calling and quality scoring of the run were processed using the manufacturer’s software RTA v3.4.4.

### 2.4 RNA extraction and sequencing

#### 2.4.1 cDNA long reads

For the complementary DNA (cDNA) long read sequencing, liquid nitrogen fresh-frozen liver, spleen and brain specimens were used for total RNA extraction using RNeasy Micro Kit (Qiagen), following the manufacturer’s protocol. The total RNA was quantified by Qubit RNA BR Assay kit (Thermo Fisher Scientific) and the RNA integrity was estimated on a Fragment Analyzer using HS RNA Kit (Agilent). The total RNA samples were stored at −80°C. Total RNA from the liver, spleen and brain was used for the PCR cDNA library preparation protocol, using the cDNA-PCR Sequencing Kit (SQK-PCS111) from ONT, following the manufacturer’s instructions. Briefly, the library preparation protocol started with 200 ng of total RNA and targeted the 3ʹ polyadenylated fraction of RNA to perform reverse transcription and strand switching with strand Switching primer (ONT) with Maxima H Minus Reverse Transcriptase (Thermo Fisher), resulting in the preparation of full-length cDNA. During the strand-switching step, a Unique Molecular Identifier was incorporated before the double-stranded cDNA was amplified by PCR with LongAmp Hot Start Taq 2X Master Mix (NEB) using primers containing rapid attachment chemistry (ONT). Subsequently, rapid sequencing adapters, Rapid Adapter T (ONT), were added to the amplified cDNA library. The sequencing runs were performed on GridION Mk1 (ONT) using a flow cell R9.4.1 FLO-MIN106D (ONT), and the sequencing data were collected for 110 h. The quality parameters of the sequencing runs were monitored in real time by the MinKNOW^TM^ platform v22.08.9, and the basecalling was performed with Guppy v6.2.11.

#### 2.4.2 RNA-seq

Additional sequencing to enhance genome annotation was provided by Illumina short-read sequencing (151 bp PE), RNA-seq. Brain samples from 35 individuals and gonad samples from 1 individual were collected for another purpose in the summer of 2019 in the same capture location. Samples were transported to the laboratory in RNAlater (Invitrogen, Thermo Fisher Scientific) and stored at −80°C. After extraction, quality control of the RNA was performed using a 2200 TapeStation (Agilent Technologies). The libraries were prepared following the TruSeq Stranded messenger RNA(mRNA) Sample Preparation Guide using the TruSeq Stranded mRNA LT Sample Prep Kit. Briefly, cDNA was randomly fragmented followed by 5ʹ and 3ʹ adapter ligation. Adapter-ligated fragments were then amplified with PCR and gel purified. Library size was quantified using 2200 TapeStation (Agilent Technologies) and quantity check was performed by qPCR following the Illumina qPCR Quantification Guide. The sequencing was performed on an Illumina NovaSeq 6000. Base calling and quality scoring of the run were processed using the manufacturer’s software, RTA and conversion to FASTQ was done with the Illumina package bcl2fastq2 v2.20.

### 2.5 Nuclear genome assembly

**Figure 2.**
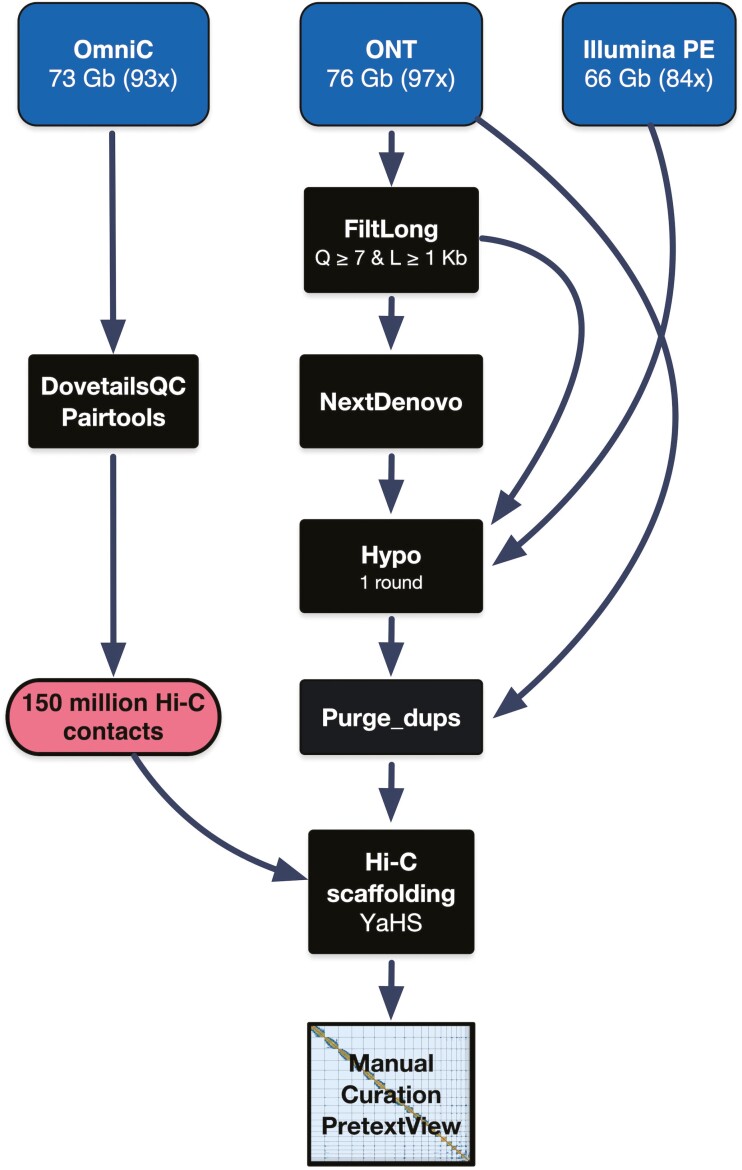
Assembly workflow. Summary of the main steps followed to obtain the curated chromosome-level assembly. See the Snakemake pipeline for detailed information (https://github.com/cnag-aat/assembly_pipeline).

The sequencing produced 75.95 Gb of ONT (96.75× coverage) and 65.61 Gb of Illumina PE 151 bp reads (83.58× coverage). These data were used with the Centre Nacional d’Anàlisi Genòmica (CNAG) snakemake assembly pipeline v1.0 (https://github.com/cnag-aat/assembly_pipeline) to obtain an optimal base assembly for further Hi-C scaffolding (a summary of the assembly workflow is shown in Fig. 2). In brief, this pipeline pre-processes the Illumina PE reads with Cutadapt^24^ v3.2 and filters the ONT reads with FiltLong v0.2.0 (https://github.com/rrwick/Filtlong), then the ONT reads are assembled with both Flye^25^ v2.9 and NextDenovo^26^ v2.4.0. The assembly pipeline also estimates genome size with GenomeScope2^27^ v2.0 by analysing the 20-mers contained in the preprocessed Illumina reads. In addition, it runs evaluations at every step, using BUSCO^28^ v5.2.2 with the vertebrata_odb10 database, Merqury^29^ v1.1 to estimate the consensus accuracy (QV) and k-mer statistics and fasta-stats.py for contiguity statistics. Our best initial assembly was obtained with NextDenovo (see details in Supplementary Table S1 and Supplementary Fig. S1). Then, the NextDenovo assembly was polished once with Illumina PE reads and ONT reads using hypo v1.0.3. Finally, the polished assembly was collapsed with purge_dups^30^ v1.2.5. Specific parameters and versions used to assemble this *X. novacula* specimen (XN0914) are detailed in Supplementary Table S2.

#### 2.5.1 Hi-C scaffolding

A total of 243.13 million Hi-C read pairs were mapped to the nextdenovo.hypo1.purged assembly (mapping statistics in Table 1) using the dovetail’s pipeline (https://omni-c.readthedocs.io/en/latest/fastq_to_bam.html) with the default minimum mapping quality threshold (−mq 40). After excluding PCR duplicates, we used 149.66 million Hi-C read pairs to scaffold the nextdenovo.hypo1.purged assembly with YaHS^31^ v1.1 with --no-contig-ec option to skip the initial contig error correction step.

**Table 1.** *Xyrichtys novacula* Omni-C mapping statistics

	Number of read pairs
Total	243,127,911
Both ends mapped	187,466,409
PCR duplicates	37,806,445
No duplicates	149,659,964
Cis	67,641,947
Trans	82,018,017
Valid (cis ≥ 1 kb + trans)	139,621,798
Cis < 1 kb	10,038,166
Cis ≥ 1 kb	57,603,781
Cis ≥ 10 kb	46,728,650

#### 2.5.2 Curation

To guide manual curation of the assembly, we computed the ONT coverage for all positions in the assembly (using Minimap2^32^ v2.18, SAMtools^33^ v1.9 and BedTools^34^ v2.29.0), as well as the location of gaps (fasta-stats.py) and telomeres with *tidk* explore (https://github.com/tolkit/telomeric-identifier) using options −x 12 −m 5. These extensions were added to the contact map using PretextGraph (https://github.com/wtsi-hpag/PretextGraph). Manual curation was performed according to the rapid curation protocol from The Sanger Institute (https://gitlab.com/wtsi-grit/rapid-curation) using PretextView (https://github.com/wtsi-hpag/PretextView).

#### 2.5.3 Decontamination

After curation, we ran the BlobToolKit INSDC pipeline,^35^ using the National Center for Biotechnology Information (NCBI) nt database (updated on June 2023) and the following BUSCO odb10 databases: actinopterygii, vertebrata, metazoa, eukaryota, fungi and bacteria.

### 2.6 Nuclear genome annotation

Repeats present in the genome assembly were annotated with RepeatMasker^36^ v4.1.2 using the custom repeat library available for *Danio rerio*. Moreover, a new repeat library specific for our assembly was made with RepeatModeler^37^ v1.0.11. After excluding repeats that were part of repetitive protein families (performing a BLAST^38^ search against Uniprot) from the resulting library, RepeatMasker was run again with this new library in order to annotate the specific repeats.

**Figure 3.**
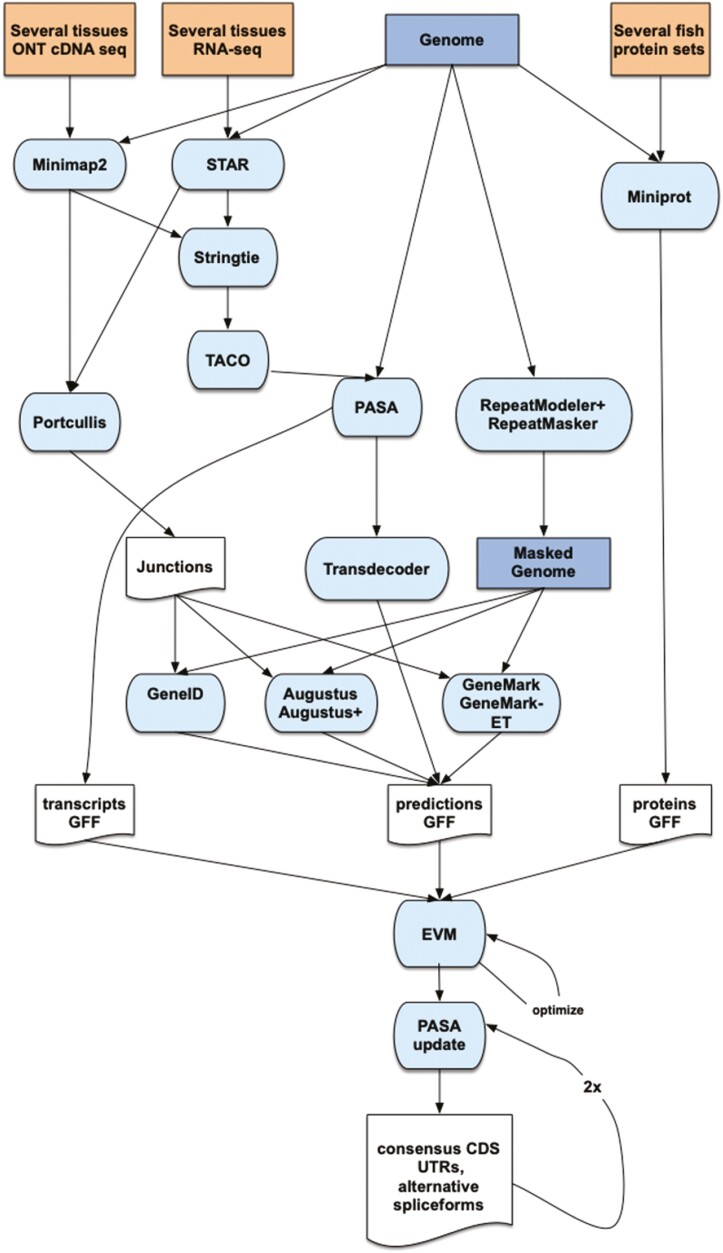
Annotation workflow. Summary of the main steps followed to annotate the *Xyrichtys novacula* genome.

The gene annotation of the assembly was obtained by combining transcript alignments, protein alignments and *ab initio* gene predictions. A flowchart of the annotation process is shown in Fig. 3. Firstly, RNA sequence information from four different tissues was obtained with both Illumina RNA-seq and ONT direct cDNA-seq. After sequencing, the long and short reads were aligned to the genome using, respectively, STAR^39^ v2.7.10a and Minimap2^32^ v2.24 with the splice option. Transcript models were subsequently generated using Stringtie^40^ v2.2.1 on each binary alignment map (BAM) file and then all the models produced were combined using TACO^41^ v0.7.3. High-quality junctions to be used during the annotation process were obtained by running Portcullis^42^ v1.2.4 after mapping with STAR and Minimap2. Finally, PASA assemblies were produced with PASA^43^ v2.5.2. The TransDecoder program, which is part of the PASA package, was run on the PASA assemblies to detect coding regions in the transcripts. Secondly, the complete proteomes of *Carassius auratus, Cynoglossus semilaevis, D. rerio, Oryzias latipes, Parambassis ranga, Sparus aurata* and *Scopthalmus maximus* were downloaded from Uniprot in March 2022 and aligned to the genome using Miniprot^44^ v0.6. *Ab initio* gene predictions were performed on the repeat-masked assembly with three different programs: GeneID^45^ v1.4, Augustus^46^ v3.5.0 and Genemark-ET^47^ v4.71 with and without incorporating evidence from the RNA-seq data. The gene predictors were run with trained parameters for human, except Genemark, which runs in a self-trained mode. Finally, all the data were combined into consensus CDS models using EvidenceModeler-1.1.1 (EVM).^43^ Additionally, UTRs and alternative splicing forms were annotated via two rounds of PASA annotation updates. Functional annotation was performed on the annotated proteins with Blast2go.^48^ First, a Blastp^38^ search was made against the NCBI nr database (last accessed March 2023). Furthermore, Interproscan^49^ v5.55_88.0 was run to detect protein domains on the annotated proteins. All these data were combined by Blast2go, which produced the final functional annotation results.

The annotation of non-coding RNAs (ncRNAs) was obtained by running the following steps. First, the program cmsearch^50^ v1.1.4 that is part of the Infernal^51^ package was run against the RFAM database of RNA families^52^ v12.0. Additionally, tRNAscan-SE^53^ v2.0.11 was run in order to detect the transfer RNA (tRNA) genes present in the masked genome assembly. Identification of long non-coding RNAs(lncRNAs) was done by first filtering the set of PASA-assemblies that had not been included in the annotation of protein-coding genes to retain those longer than 200 bp and not covered more than 80% by a small ncRNA. The resulting transcripts were clustered into genes using shared splice sites or significant sequence overlap as criteria for designation as the same gene.

### 2.7. Mitogenome assembly and annotation

We employed a strategy that uses a reference bait to first capture mitochondrial nanopore reads, assembles them into a single circular contig and then polishesthe assembly with Illumina short reads (Supplementary Fig. S2). To obtain the mitochondrial sequences, all ONT reads previously filtered for whole-genome assembly with FiltLong v.0.2.1 (https://github.com/rrwick/Filtlong) to be at least 1 Kb long and have a mean quality of 7 were mapped with Minimap2^32^ v 2.24 against the linear complete mitochondrial genome of *Thalassoma lunare* (NCBI accession NC_048980 and length 17,073 bp) with option: ‘-ax map-ont’. We retained all reads with mapping quality = 13 (relatively unique) and at least 8,000 exact matches to the mitochondrial genome reference; these included 297 reads and a total of 4,883,504 bp (estimated mitochondrial coverage for a 25 Kb genome 195×).

All the retained ONT reads were assembled with Flye^25^ v2.9 using the options: ‘flye --meta --scaffold -i 2 -g 25k --nano-raw’. The --meta option is the most appropriate for uneven coverage samples and -i 2 specifies two polishing iterations with the ONT reads on the final assembly with. The output assembly consists in a single circular contig with coverage ≥ 249× and length 17,293 bp. Note that its length is similar to both, the *T. lunare* mitogenome (used to select the ONT reads) and our previous mitogenome assembly obtained with Illumina and accession MN794015.1.^54^

Before polishing, we mapped all the Illumina PE reads to the Flye long-read assembly using BWA-MEM^55^ v0.7.17-r1188. The alignment was subsequently filtered with samtools^33^ v1.15.1 to discard PCR duplicates and keep mapped read pairs to the circular contig as follows: *samtools view -F 1024 -f 3 -L circular_contig.bed > Illumina_mito.bam*. These read pairs were pulled out from the alignment using *samtools fastq*. A total of 177,280 read pairs were collected for further polishing (estimated coverage at least 2,130.14×). In order to improve the sequence accuracy of the assembled mitochondrial genome, we performed two additional rounds of polishing on the circular contig with the selected Illumina reads using NextPolish^56^ v1.4.1 with options: ‘-paired -max_depth 1000’.

Finally, the polished circular contig was rotated and oriented as follows. First, we annotated the contig using MITOS^57^ v2.1.3 with parameters *-c 2 --linear --best -r refseq81m*. Second, we used the coordinates in the results.bed file to orient the mitogenome using a perl script (https://github.com/cnag-aat/FOAM/blob/main/scripts/orient_mitogenome_v1.pl), that ensures it starts with the conventional tRNA Phenyl-Alanine (*trnF*) (e.g.^58^). For evaluation, we first rotated the previous Illumina mitogenome in NCBI (MN794015.1) using the same approach ensuring they start at the same gene. Then we used *dnadiff* from mummer package^59^ v3.23 to align our new mitogenome assembly against the rotated MN794015.1 and Merqury^29^ v1.3 to estimate the QV using the selected mitochondrial Illumina reads.

## 3. Results and discussion

### 3.1. Nuclear genome assembly

**Figure 4.**
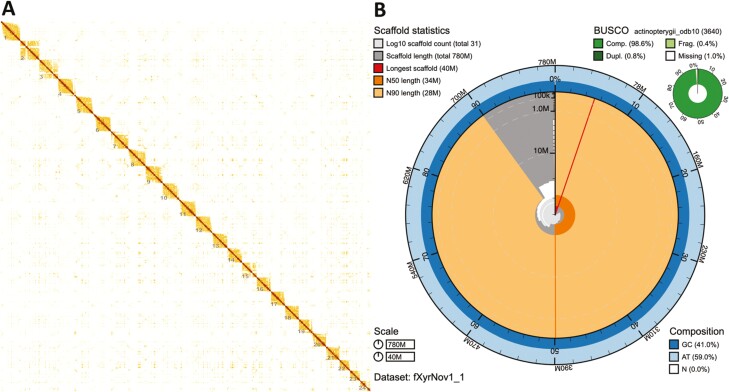
Summary of assembly results. (A) Hi-C contact map for genome assembly fXyrNov1_1 visualized in PretextView. The map shows 24 superscaffolds, ordered from longest to shortest, in agreement with the karyotype of the species (*n* = 24). (B) Snail plot summary of assembly statistics for assembly fXyrNov1_1. The main plot is divided into 1,000 size-ordered bins around the circumference with each bin representing 0.1% of the 775,535,542 bp assembly. The distribution of scaffold lengths is shown in dark grey with the plot radius scaled to the longest scaffold present in the assembly (40,352,512 bp, shown in red). Orange and pale-orange arcs show the N50 and N90 scaffold lengths (34,328,652 and 28,232,734 bp), respectively. The pale grey spiral shows the cumulative scaffold count on a log scale with white scale lines showing successive orders of magnitude. The blue and pale-blue area around the outside of the plot shows the distribution of GC, AT and N percentages in the same bins as the inner plot. A summary of complete, fragmented, duplicated and missing BUSCO genes in the actinopterygii_odb10 set is shown in the top right.

Given the high quality and contiguity of the assembly obtained from YaHS, the curation was minimal, comprising 2.58 interventions per Gb (i.e. only 17 edits). The decontamination analysis confirmed that all 31 scaffolds matched the phylum Chordata in the database (Supplementary Fig. S3), showing no evidence for contamination in the assembly. The identifier of our final (curated and decontaminated) genome assembly is fXyrNov1.1. It has scaffold N50 of 34,328,652 bp (*n* = 11), N90 of 21,814,093 (*n* = 21), 25 gaps and a span of 775,535,142 bp. In fact, the assembly span is very close to the GenomeScope estimate of 784.38 Mb. Moreover, 99.63% of the sequence is contained in 24 superscaffolds, which is consistent with a haploid chromosome number of 24. In fact, 5 superscaffolds contain telomeres at both ends, and the rest contain telomeres at least at one end (Supplementary Tables S3 and S4). Finally, it has a QV = 42.92, low levels of false duplications (Supplementary Table S1 and Supplementary Fig. S4) and its gene completeness estimated using BUSCO^28^ v5.3.2 with actinopterygii_odb10 is C:98.6%[S:97.9%,D:0.7%],F:0.4%,M:1.0%,n:3640 (Fig. 4 and Supplementary Table S1).

### 3.2. Nuclear genome annotation

In total, we annotated 26,690 protein-coding genes that produce 43,547 transcripts (1.63 transcripts per gene) and encode 39,613 unique protein products. We were able to assign functional labels to 93.3% of the annotated proteins. The annotated transcripts contain 11.82 exons on average, with 93% of them being multi-exonic (Table 2). In addition, 12,737 non-coding transcripts were annotated, of which 10,450 and 2,287 are long and short ncRNA genes, respectively.

**Table 2.** *Xyrichtys novacula* genome annotation statistics

	XNOV1A
Number of protein-coding genes	26,690
Median gene length (bp)	7,733
Number of transcripts	43,547
Number of exons	281,303
Number of coding exons	263,943
Median UTR length (bp)	1,895
Median intron length (bp)	345
Exons/transcript	11.82
Transcripts/gene	1.63
Multi-exonic transcripts	93%
Gene density (gene/Mb)	34.41

### 3.3. Mitogenome assembly and annotation

The mitogenome, fXyrNov1.1_MT, consists in one single circular contig 17,298 bp long and has a QV of 42.38. Its annotation contains 38 exons, 13 protein-coding genes, 13 mRNAs, 24 ncRNA genes, 1 origin of replication, 2 ribosomal RNAs (rRNA) and 22 tRNAs. Finally, an alignment against the previous Illumina assembly (NCBI accession NC_048980)^54^ is completely collinear, with a sequence identity of 99.63% (53 single nucleotide polymorphisms, SNPs) (Supplementary Fig. S5). Our new mitogenome assembly corresponds to a different specimen and contributes to enrich the sequence diversity in public databases.

## 4. Conclusions

In this study, we present the chromosome-level assembly and annotation of the *X. novacula* genome, which opens new opportunities for research in various fields. Our results confirm a haploid chromosome number of 24 and a lack of sexual chromosomes in this species.^20,21,23^ Furthermore, the high-quality genome annotation presented here can serve to delve into diverse aspects of the biology and ecology of this species from a molecular perspective.

Studies on the reproductive biology of *X. novacula* can be of particular interest due to its apparent lack of sexual chromosomes and its sequential hermaphroditism mediated by environmental factors. The cascade of molecular and physiological changes associated with sex transitioning in fish are still under study,^13^ we believe that providing high-quality genomic information on more species can be crucial for future research in this field. Additionally, populations of this species can be easily monitored in the laboratory and in the wild.^10,16^ The combination of high-resolution acoustic telemetry, a powerful tool that allows the study of fish populations in their natural habitat,^60^ and cutting-edge genomics can result in significant advancements in the fields of behavioural, molecular and conservation biology.^61^ Therefore, we strongly believe that *X. novacula* represents an exceptional candidate for conducting such multidisciplinary comprehensive studies.

Like other coastal species with similar distribution, *X. novacula* faces significant human pressure, particularly for recreational purposes including fishing.^62,63^ In fact, in the Balearic Islands, this species is a major target in recreational fisheries, leading to the implementation of specific conservation measures aimed to protect its populations.^12^ Traditionally, conservation efforts to preserve marine fauna have focussed on ecological and biometrical factors (e.g. individual size), often overlooking the genetic consequences of human pressures on populations. However, the exploitation of fish populations can result in genetic diversity loss.^64^ Marine protected areas have been proposed as valuable management tools to protect genetic diversity and serve as reservoirs of susceptible genotypes.^19^ Understanding the impact of fishing on fish populations and the role of marine protected areas from a molecular perspective is crucial for the efficient management and preservation of fish communities.^64^

Having a reference genome available significantly expands the scope and potential impact of studies conducted on this species. It provides a solid foundation for genomic research into the molecular basis of relevant biological processes and on the effects of fishing on the genetics and epigenetics of coastal fish populations, enabling informed managerial decisions based on scientific evidence. Overall, our contribution allows for further exploration of *X. novacula*’s biology, offering valuable insights that benefit not only this species but also other species sharing similar biological characteristics and facing comparable human pressures.

## Supplementary Material

dsad021_suppl_Supplementary_Tables_S1-S3

dsad021_suppl_Supplementary_Figures_S1-S3

## Data Availability

All the data generated in this study, including sequencing reads and annotated assembly, are available at the European Nucleotide Archive (https://www.ebi.ac.uk/ena/browser/home) under the Project accession numbers PRJEB56817 and PRJEB65970. In addition, a genome browser and annotation data can be found at https://denovo.cnag.cat/raor. A Snakemake pipeline for genome assembly is available at https://github.com/cnag-aat/assembly_pipeline.
