# Supplementary material for: Chromosome-level assembly and annotation of the Xyrichtys novacula (Linnaeus, 1758) genome
Source: DNA Res. 2023 Oct 5;30(5):dsad021. doi: 10.1093/dnares/dsad021 (PMC10590160; doi:10.1093/dnares/dsad021)
Supplement: dsad021_suppl_Supplementary_Figures_S1-S3 [file dsad021_suppl_supplementary_figures_s1-s3.docx]

# **Supplementary figures**

Chromosome-level assembly and annotation of the *Xyrichtys novacula* (Linnaeus, 1758) genome

**Running title:**

Chromosome-level genome assembly of *Xyrichtys novacula*

**Authors:**

Fernando Cruz^1^, Jèssica Gómez-Garrido^1^, Marta Gut^1^, Tyler S. Alioto^1^, Joan Pons^2^, Josep Alós^2^, Margarida Barcelo-Serra^2*^

**Affiliations:**

^1^Centro Nacional de Análisis Genómico (CNAG), C/Baldiri Reixac 4, 08028 Barcelona, Spain

^2^Institut Mediterrani d’Estudis Avançats, IMEDEA (UIB-CSIC), C/ Miquel Marquès 21, 07190 Esporles, Illes Balears, Spain

*Corresponding author: Margarida Barcelo-Serra ([mbarcelo@imedea.uib-csic.es](mailto:mbarcelo@imedea.uib-csic.es)). C/ Miquel Marquès, 21, 07190 Esporles, Illes Balears Spain. Phone (+34)971611950 Fax. (+34)971611761.


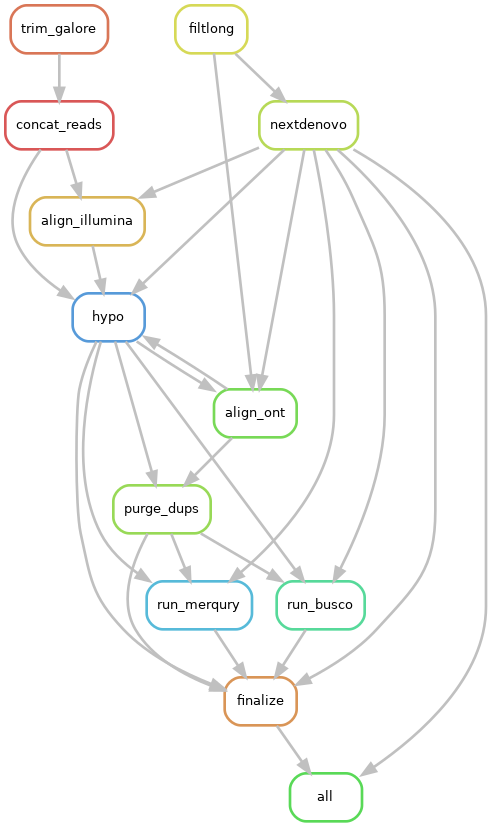


Supplementary Figure S1

NextDenovo rule graph of the *Xyrichtys novacula* genome assembly process for assembly XyrNov1_1.


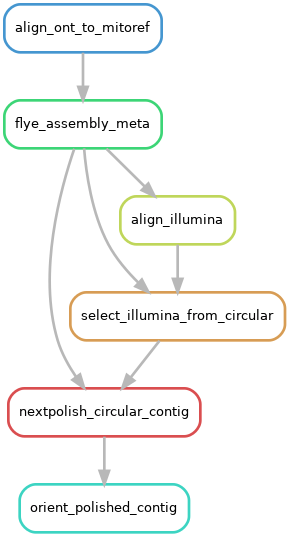


Supplementary Figure S2

NextDenovo rule graph workflow of the snakemake pipeline used to assemble de *Xyrichtys novacula* mitogenome (fXyrNov1.1_MT). Pipeline v0.2 available in github: <https://github.com/cnag-aat/FOAM>


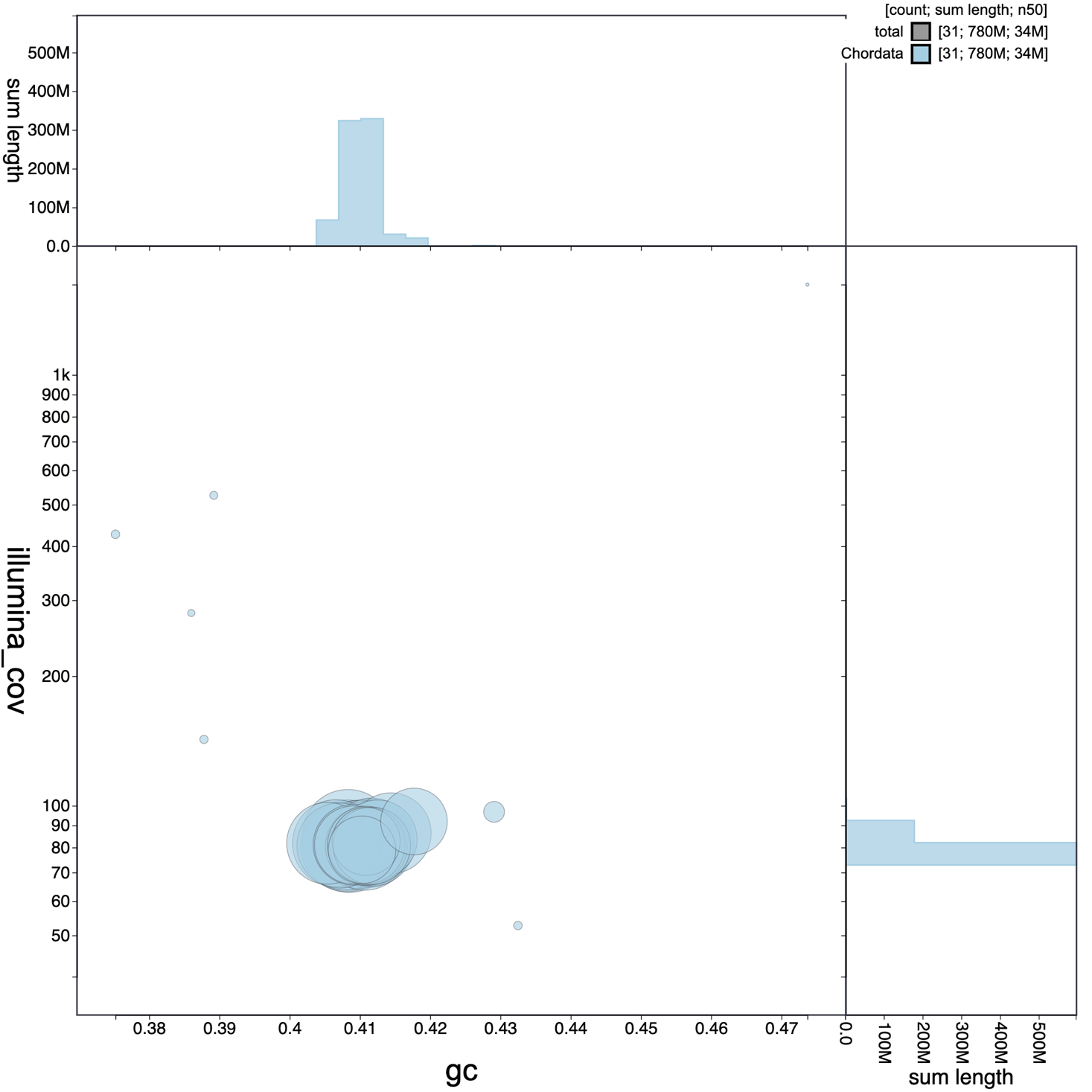


Supplementary Figure S3

Blob plot of base coverage in Illumina against GC proportion for scaffolds in assembly fXyrNov1_1. Scaffolds are colored by phylum. Circles are sized in proportion to scaffold length. Histograms show the distribution of scaffold length sum along each axis.


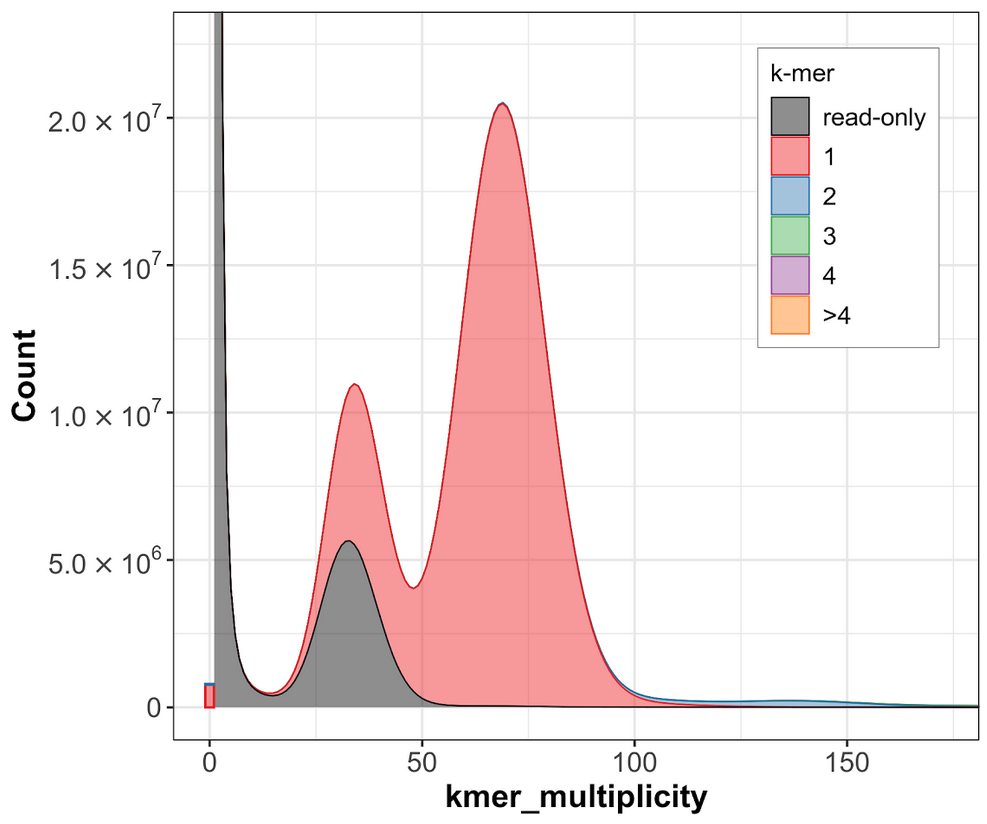


Supplementary Figure S4

Stacked histogram of fXyrNov1.1 obtained with Merqury by comparing 20-mers in the assembly to those contained in the Illumina reads. The blue line shows 20-mers appearing twice in the assembly. The levels of artificial duplications are negligible, they correspond to the blue line above the homozygous peak at 68x.


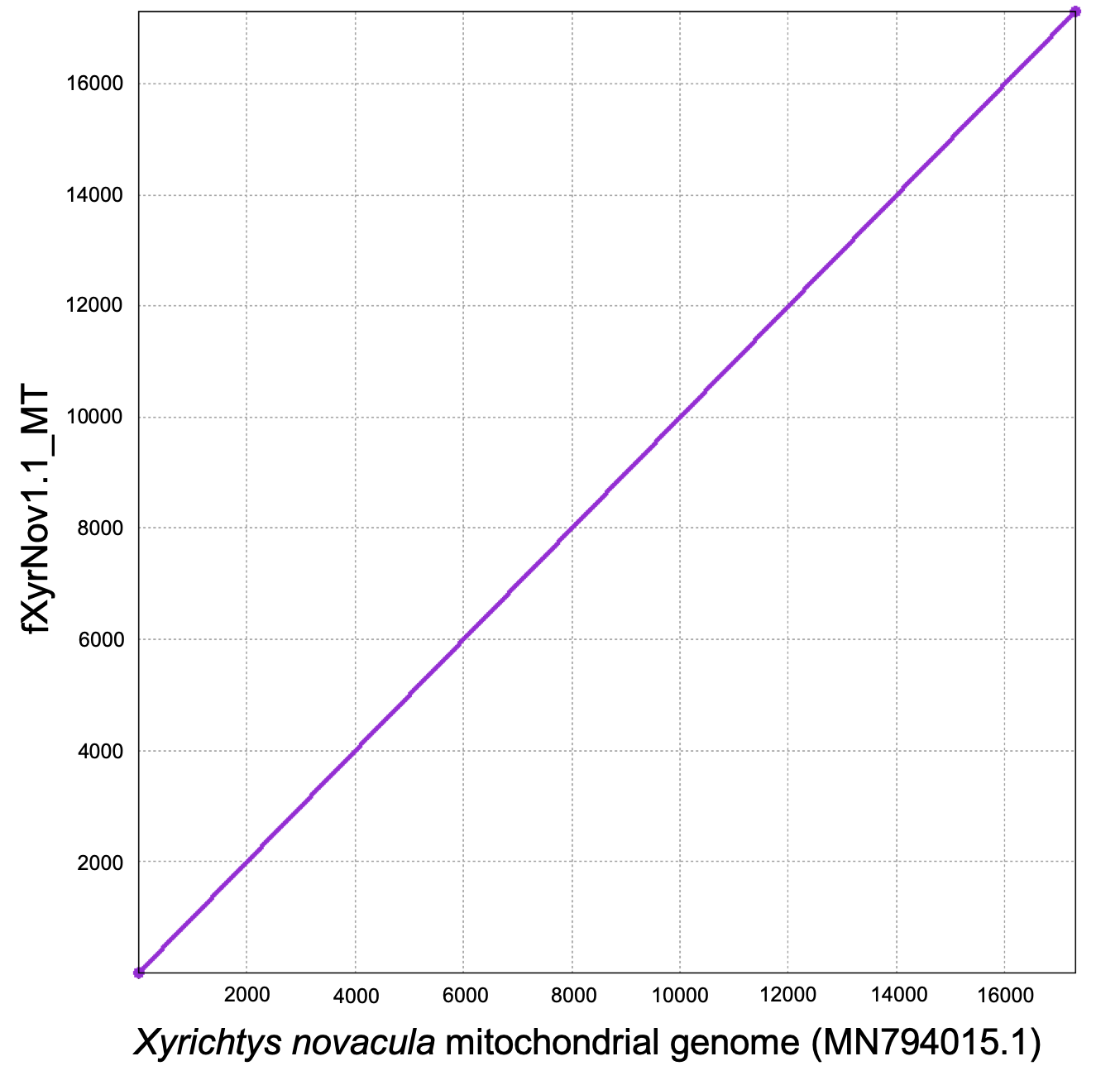


Supplementary Figure S5

Alignment of the mitogenome assembly obtained in this study (fXyrNov1.1_MT) and the mitogenome previously reported for *Xyrichtys novacula*, accession number MN794015.1 [(Barcelo-Serra et al. 2020)](https://www.zotero.org/google-docs/?ZkHc1M)*.

* Barcelo-Serra M, Pons J, Viver T, Rosselló-Mora R, Alós J. 2020. Complete mitochondrial genome of the pearly razorfish *Xyrichtys novacula*: phylogenetic analysis of its placement within the Labridae family. Mitochondrial DNA Part B. 5(1):644–645. doi:10.1080/23802359.2019.1711226.
